# Mapping the healing brain: Longitudinal MRI volumetrics and outcomes across surgical techniques for primary brain abscesses

**DOI:** 10.1016/j.bas.2026.105942

**Published:** 2026-01-11

**Authors:** Biyan Nathanael Harapan, Antonia Clarissa Wehn, Janine Herrmann, Béatrice Grabein, Florian Ringel, Michael Schmutzer-Sondergeld

**Affiliations:** a Department of Neurosurgery, LMU University Hospital, LMU Munich, Marchioninistraße 15, 81377, Munich, Germany; b Department of Clinical Microbiology and Hospital Hygiene, LMU University Hospital, LMU Munich, Marchioninistraße 15, 81377, Munich, Germany

**Keywords:** Brain abscess, Intracranial abscess, Craniotomy, Stereotaxy, Infection, Neurosurgery

## Abstract

**Introduction:**

Primary intracerebral abscesses are rare but life-threatening infections requiring prompt surgical and antibiotic treatment. Comparative outcome data on neurosurgical techniques and radiological evolution remain limited.

**Research question:**

Do clinical outcomes and MRI-based volumetric changes differ between stereotactic aspiration, craniotomy, and burr-hole trepanation in adults and children with primary intracerebral abscesses?

**Material and methods:**

We retrospectively reviewed surgically treated patients between 2014 and 2024 at the LMU University Hospital in Munich. Abscess and perilesional edema volumes were quantified on serial MRI at clinically defined follow-up intervals. Clinical outcomes were assessed using standardized neurological and functional scales, and recurrence was further evaluated. Adult and pediatric subgroups were analyzed separately.

**Results:**

Sixty patients underwent stereotactic aspiration (53.3 %), craniotomy (36.7 %), or burr-hole trepanation (10.0 %). Mean abscess volume decreased from 18.8 cm^3^ preoperatively to 10.8 cm^3^ postoperatively, 4.4 cm^3^ at 4–12 weeks, and 2.2 cm^3^ at final follow-up. Edema volume declined from 53.4 cm^3^ to 35.8 cm^3^ postoperatively, 10.6 cm^3^ at 4–12 weeks, and 3.5 cm^3^ at last follow-up. Volume reduction patterns were similar across surgical approaches, and no significant volumetric differences were observed between pediatric and adult patients. Recurrence was unrelated to surgical modality.

**Discussion and conclusion:**

All three surgical approaches achieved substantial and sustained reductions in abscess and edema volumes, with comparable neurological outcomes across age groups. Serial MRI volumetrics provide detailed insight into the temporal evolution of intracerebral abscesses and may inform postoperative monitoring and follow-up strategies for primary brain abscesses.

## Highlights

• Longitudinal MRI volumetrics characterize abscess and edema regression after surgery.
• Stereotaxy, craniotomy, and burr-hole trepanation showed comparable abscess volume reduction.
• Craniotomy favored for cortical abscesses; stereotaxy for deep-seated lesions.
• Shorter intravenous antibiotics (≤6 weeks) did not affect functional outcome.
• Serial MRI may support postoperative monitoring in primary brain abscess care.

## 1 Introduction

Intracerebral abscesses are uncommon but potentially fatal infections of the brain parenchyma (Muzumdar et al., 2011). Etiologically, most intracerebral abscesses are bacterial in origin, with streptococci being the most frequently isolated pathogens (Sonneville et al., 2017). However, polymicrobial infections are common (Eichorn et al., 2025), particularly in patients with prior otorhinolaryngological procedures, dental infections, or underlying immunosuppression due to malignancies or HIV/AIDS (Eichorn et al., 2024). Their management is complex and typically requires coordinated multidisciplinary care (Omland et al., 2024), since timely neurosurgical intervention combined with empirically started – and later pathogen-directed – antimicrobial therapy remains central to achieve favorable outcomes (Brouwer et al., 2014). Surgical options (for example, stereotactic aspiration, open craniotomy or burr-hole drainage) are chosen according to lesion size, localization and clinical status and are complemented by prolonged antibiotic courses and source control measures (Penezic et al., 2021; Alvis Miranda et al., 2013). Early drainage to reduce mass effect and to obtain microbiological specimens, together with targeted antimicrobial therapy, continue to form the backbone of contemporary treatment algorithms.

The clinical presentation is often nonspecific, ranging from headache, fever, and focal neurological deficits to signs of elevated intracranial pressure or reduced consciousness (Huang et al., 2021).

While both stereotactic and open surgical techniques are routinely used in clinical practice, there is limited comparative evidence regarding their respective outcomes in terms of clinical recovery, recurrence rates, and radiological resolution (Ali et al., 2023; Tan et al., 2010). Particularly, the evolution of abscess and edema volumes on magnetic resonance imaging (MRI), as well as their relationship to symptom improvement and long-term outcome, remains insufficiently studied in a real-world clinical setting. Most available studies focus on dichotomous clinical endpoints such as mortality or recurrence, while systematic, longitudinal quantitative MRI volumetric assessments are scarce (Eibl et al., 2025; Liebert et al., 2025; Murakami et al., 2025). Moreover, comparative data evaluating how different surgical approaches influence the temporal pattern of abscess and edema resolution are largely lacking. Addressing these gaps is essential for informing postoperative monitoring, interpreting follow-up imaging, and guiding timely clinical decision-making.

Therefore, this study aims to compare clinical and radiological outcomes, utilizing longitudinal MRI volumetrics, among patients undergoing stereotactic aspiration, craniotomy, and burr-hole trepanation for primary intracerebral abscesses.

## 2 Material and methods

### 2.1 Study design and objectives

**Fig. 1:**
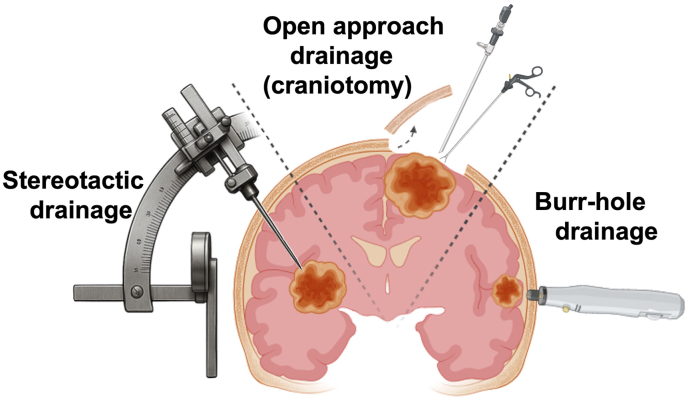
Schematic illustration of the three principal neurosurgical approaches employed for abscess evacuation: stereotactic aspiration, open craniotomy, and burr-hole trepanation. Each technique is depicted in a simplified manner to highlight the different surgical access routes for intracerebral abscess drainage.

This retrospective, observational study aims to evaluate the clinical and radiological outcomes of patients treated for primary intracerebral abscesses between 01/2014 and 12/2024 at the LMU University Hospital in Munich. The study focuses on the effectiveness and tolerability of previous therapeutic approaches, with particular attention to postoperative symptom resolution or improvement, as well as potential associations between surgical techniques – specifically stereotactic aspiration, open craniotomy or burr-hole trepanation – and clinical and radiological outcome (see Fig. 1).

### 2.2 Patient selection and inclusion criteria

Patients were included if they were diagnosed with a primary intracerebral abscess, had no prior history of neurosurgical intervention, and received surgical treatment at the LMU University Hospital in Munich. Only patients with available follow-up clinical and imaging data were considered. Patients were excluded if these criteria were not met. The study size reflects all eligible patients treated surgically for primary intracerebral abscesses at our institution during 2014–2024.

### 2.3 Data collection and parameters

All clinical and imaging data were retrieved retrospectively from the institutional databases. Pre- and postoperative MRI imaging, acquired as part of routine clinical care, were reviewed to evaluate abscess and perilesional edema volumes. Furthermore, a comprehensive set of clinical parameters was collected to characterize patient status at baseline and during follow-up. These included the Glasgow Coma Scale (GCS) at admission/preoperatively and immediately postoperatively, symptomatology before and after surgery, Karnofsky Performance Status (KPS) at admission and immediately postoperatively, the modified Rankin Scale (mRS) at both timepoints, and the Glasgow Outcome Scale (GOS) immediately postoperatively. Additional data included the date of surgery, antibiotic treatment regimen, number of brain abscesses, number and species of causative pathogens, presumed infectious source (focus), total duration of antibiotic therapy, duration of intravenous and oral antibiotic administration, and the number of antibiotic regimen changes due to clinical or microbiological adjustments.

The presence of immunosuppression (yes/no) and dexamethasone therapy (yes/no) was recorded, with dexamethasone exposure categorized as follows: 0 = no dexamethasone, 1 = up to 3 days, 2 = 4 days to 1 week, 3 = 1–2 weeks, 4 = 2–3 weeks, and 5 = more than 3 weeks. Body mass index (BMI) was also documented.

In addition, patient frailty was assessed using the Modified 5-Item Frailty Index (mFI-5), which includes the following variables: (1) history of severe chronic obstructive pulmonary disease (COPD), (2) congestive heart failure (CHF), (3) preoperative functional status (independent vs. partially or completely dependent), (4) hypertension requiring medication, and (5) diabetes mellitus treated with oral agents or insulin. Each item was assigned one point, resulting in a total score ranging from 0 to 5. Patients were classified into three frailty categories: mFI = 0 (not frail), 1 (mild frailty), mFI = 2 (moderate frailty), and mFI ≥3 (severe frailty) (Chung et al., 2024).

A subset of patients underwent re-operation for recurrent or persistent intracerebral abscesses. Recurrence was defined as the need for additional surgical intervention following an initial procedure due to radiological evidence of renewed abscess growth or insufficient resolution on follow-up MRI, typically accompanied by persistent or worsening neurological symptoms despite ongoing antimicrobial therapy. Persistence was defined as radiological stagnation or insufficient reduction of abscess volume on early postoperative MRI, most commonly within the first 2–4 weeks after surgery, prompting re-intervention despite appropriate antibiotic treatment.

Decisions regarding re-operation were made on an individual basis through interdisciplinary consensus involving neurosurgery, neuroradiology, and infectious disease specialists. Indications for re-intervention included lack of expected volumetric reduction on serial MRI, increase in abscess size, new or progressive neurological deficits, or failure of clinical improvement under adequate antimicrobial therapy.

### 2.4 Magnetic resonance imaging

**Fig. 2:**
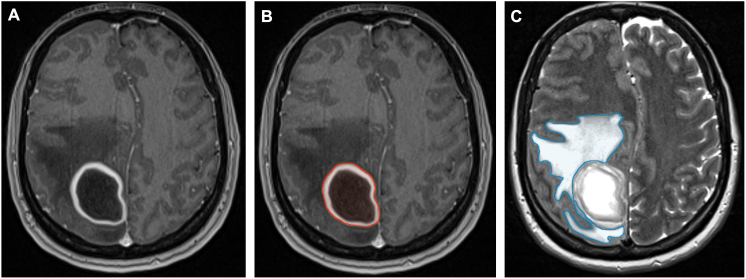
Representative cranial MRI sequences of a brain abscess. A: Axial T1-weighted contrast-enhanced sequence demonstrating a right parietal brain abscess. B: Segmentation of abscess volume (outlined in red) on contrast-enhanced T1-weighted MRI. C: Segmentation of perilesional edema volume (outlined in blue) on T2-weighted MRI.

Preoperative and postoperative cranial MRI (cMRI) examinations were conducted using either 1.5- or 3.0-T scanners (Magnetom Symphony, Siemens, Erlangen, Germany; Signa HDxt, GE Healthcare, Little Chalfont, United Kingdom). Standard imaging protocols included axial T2-weighted sequences with a slice thickness of 2 mm, as well as three-dimensional T1-weighted sequences acquired before and after intravenous administration of gadopentetate dimeglumine (0.1 mmol/kg body weight; Magnevist, Schering Corporation, Kenilworth, NJ). Each sequence was reconstructed in axial, sagittal, and coronal planes. Volumetric assessment of abscesses and perilesional edema on pre- and postoperative MR images was performed via semi-manual segmentation of contrast-enhanced T1-and T2-weighted sequences using the SmartBrush® tool (SmartBrush®, Elements®, BRAINLAB AG, Munich, Germany). For the volumetric analysis, the entire abscess was meticulously outlined in each slice of contrast-enhanced T1-weighted MRI sequence. For abscess volumetrics, segmentation encompassed the entire lesion, including both the contrast-enhancing rim and the non-enhancing central portion, without differentiating between these components in the final calculation. The contrast enhancement intensity itself was not considered relevant for delineation. The boundary was defined at the exact interface where the contrast-enhancing rim terminated adjacent to normal parenchyma, with parenchymal signal intensity matching that of uninvolved brain tissue (see Fig. 2A and B). Perilesional edema was segmented on T2-weighted sequences (Fig. 2 C). In patients with multiple intracerebral abscesses, all individual abscess cavities were segmented at each imaging time point, and the cumulative sum of these volumes was used as the patient-level abscess volume for analysis. Perilesional edema was assessed analogously by summing the edema volumes surrounding each abscess, thereby reflecting the total intracranial infectious burden.

This standardized segmentation protocol was applied uniformly across all time points to enable precise longitudinal comparisons. Representative examples of abscess and edema segmentation are shown in Fig. 2A–C. All volumetric measurements were performed blinded to the MRI time points by two independent investigators (B.N.H. and M.S.-S.). Each investigator completed the full segmentation workflow individually for all images, and the final abscess and edema volumes were calculated as the average of both raters’ results.

Postoperative MRI follow-up intervals were determined by clinical indication rather than a predefined imaging protocol. While early postoperative imaging was routinely obtained within the first five days after surgery, subsequent MRI examinations were scheduled based on clinical evolution, laboratory parameters, and neurological status. As a result, intermediate follow-up scans were heterogeneous and most frequently clustered either within the first four weeks or within a broader 4–12 week postoperative window. The limited number of patients with imaging at identical predefined time points (e.g., exactly 2, 4, 8, or 12 weeks) precluded statistically meaningful volumetric comparisons at these discrete intervals. Accordingly, volumetric changes were analyzed across clinically relevant time windows reflecting real-world practice rather than fixed biological time points.

No predefined volumetric thresholds were established to mandate re-intervention. Interpretation of follow-up MRI findings and assessment of expected abscess volume reduction were performed at the discretion of the treating neurosurgeon within an interdisciplinary decision-making framework, integrating radiological evolution with clinical status.

### 2.5 Treatment protocol

Surgical interventions included stereotactic abscess aspiration, craniotomy or burr-hole trepanation for abscess evacuation. In stereotactic procedures, surgical planning was performed using the iPlan Stereotaxy software (Brainlab, Munich, Germany), based on stereotactically localized contrast-enhanced computed tomography (CT) scans with a slice thickness of 0.6 mm, co-registered with preoperative MRI data sets (contrast-enhanced T1-weighted sequences, T2-weighted sequences, and contrast-enhanced magnetic resonance angiography) (Thorsteinsdottir et al., 2025). The Leksell® Coordinate Frame G (Elekta GmbH, Hamburg, Germany) was utilized to ensure precise stereotactic navigation. Through a 2 mm burr hole, a 1.0 mm biopsy/aspiration guide tube (inomed Medizintechnik GmbH, Emmendingen, Germany) was stereotactically inserted for targeted abscess access. Repeated irrigation with sterile sodium chloride solution was performed to facilitate effective abscess lavage (Schmutzer-Sondergeld et al., 2024a, 2024b). The choice of surgical approach was made individually for each patient by consensus among experienced neurosurgeons, based on abscess configuration, volume, clinical presentation, and patient-specific risk assessment. The duration and regimen of antibiotic therapy were determined interdisciplinarily through collaboration between our neurosurgical team and the hospital's antibiotic stewardship team. Decisions were based on abscess characteristics, microbiological findings, clinical course, and follow-up imaging.

### 2.6 Statistical analysis

All statistical analyses were performed using established statistical software GraphPad Prism 8.0 (GraphPad, San Diego, CA, USA). After assessment for normal distribution, continuous variables were compared using Student's *t*-test or the Mann–Whitney *U* test as appropriate. For comparisons involving more than two groups with small and unequal sample sizes, continuous variables were analyzed using the non-parametric Kruskal–Wallis test instead of analysis of variance (ANOVA). Categorical variables, including abscess characteristics such as localization, side, and depth, were analyzed using Chi-square tests or Fisher's exact test where appropriate. Kaplan–Meier survival analyses were applied to visualize time to recurrence. Univariate and multivariate regression analyses were used to identify predictors of clinical and radiological outcomes. Multivariate analyses were performed to explore associations between clinical, radiological, and treatment-related variables and selected outcomes. Binary outcomes, including recurrence and need for revision surgery, were analyzed using multivariate logistic regression models, while continuous volumetric outcomes were examined using multivariate linear regression. Covariates were selected a priori based on clinical relevance and existing literature rather than automated selection procedures, in order to minimize overfitting. Variables entered into the models – among others – included age, sex, abscess location, preoperative abscess volume, presence of multiple abscesses, surgical approach, and dexamethasone use. Multivariate analyses were conducted with exploratory intent and interpreted cautiously in light of the retrospective design and potential residual confounding. For correlation analyses, Pearson's or Spearman's correlation coefficients were used as appropriate based on data distribution. A two-sided *p*-value of less than 0.05 was considered statistically significant.

### 2.7 Ethical considerations and data protection

Ethical approval for this retrospective study was granted by the Ethics Committee of LMU Munich (Ethikkommission der LMU München) under the project number 25–0542, with confirmation received on July 25, 2025. All data were collected in accordance with applicable data protection laws. Personal identifiers were irreversibly anonymized, and all data were stored in a secure electronic database within the hospital network. Imaging data used in this study were obtained during standard clinical procedures and interpreted at the time of treatment by the Department of Neuroradiology; all study-relevant information was extracted from finalized radiology reports.

## 3 Results

### 3.1 Patient characteristics

Between 2014 and 2024, a total of 60 patients with primary intracerebral abscesses were treated surgically at our institution. The cohort consisted of 42 males (70.0 %) and 18 females (30.0 %), with a mean age of 48.0 ± 22.7 years. Ten patients (16.7 %) were under 18 years of age, while 50 patients (83.3 %) were adults (Table 1). Surgical approaches included stereotactic aspiration in 32 patients (53.3 %), open craniotomy in 22 cases (36.7 %), burr-hole trepanation in 6 patients (10.0 %). In total, 94 surgical interventions were performed: 60 initial surgeries, with 26 second-look procedures, 5 third surgeries, and 2 fourth interventions.

**Table 1:** Baseline demographics, clinical characteristics, abscess features, and treatment-related parameters of pediatric (<18 years) and adult (≥18 years) patients with primary intracerebral abscesses.

	Age <18 yrs	Age ≥18 yrs	*p-value*
***Demographics***			
**Number of patients, n (%)**	10 (16.7)	50 (83.3)	
**Sex, n (%)**			
Male	4 (40.0)	38 (76.0)	0.05
Female	6 (60.0)	12 (24.0)	
**mFI-5, n (%)**			
Not frail		26 (52.0)	
Mild frailty		14 (28.0)	
Moderate frailty		8 (16.0)	
Severe frailty		2 (4.0)	
***Abscess characteristics***			
**Localization, n (%)**			
Frontal	6 (50.0)	21 (34.4)	0.3
Parietal	1 (8.3)	8 (13.1)	0.99
Temporal	3 (25.0)	18 (29.5)	0.99
Occipital	0	3 (4.9)	0.99
Brainstem	0	6 (9.8)	0.6
Cerebellum	2 (16.7)	5 (8.2)	0.3
**Side, n (%)**			
Right	7 (70.0)	19 (36.5)	0.08
Left	3 (30.0)	29 (55.8)	0.2
Bilateral	0	2 (3.8)	0.99
Midline	0	2 (3.8)	0.99
**Depth, n (%)**			
Cortical	8 (80.0)	24 (48.0)	0.09
Subcortical	0	23 (46.0)	**0.009**
Cortical + subcortical	2 (20.0)	3 (6.0)	0.2
**Enlarged ventricular system, n (%)**			
Yes	2 (20.0)	4 (8.0)	
No	8 (80.0)	46 (92.0)	0.3
**Presumed focus, n (%)**			
Focus unknown	0	8 (16.0)	0.3
Sinusitis	5 (50.0)	6 (12.0)	**0.01**
Otogenic	3 (30.0)	4 (8.0)	0.08
Odontogenic	1 (10.0)	15 (30.0)	0.3
Pneumogenic	0	2 (4.0)	0.99
Cardiogenic	1 (10.0)	2 (4.0)	0.4
Sepsis	0	8 (16.0)	0.3
Others	0	5 (10.0)	0.6
***Treatment***			
**Initial surgical approach, n (%)**			
Stereotaxy	3 (30.0)	29 (58.0)	0.2
Craniotomy	5 (50.0)	17 (34.0)	0.5
Burr-hole trepanation	2 (20.0)	4 (8.0)	0.3
**Duration of antibiotics (days), mean ± SD**			
total duration	65.6 ± 14.3	70.5 ± 34.5	0.7
i.v.	56.0 ± 9.7	42.6 ± 19.9	0.05
p.o.	9.6 ± 12.5	28.2 ± 32.6	0.09
**Number of antibiotic changes, mean ± SD**	2.4 ± 1.0	2.4 ± 1.4	0.9

mFI-5 = Modified 5-Item Frailty Index; SD = standard deviation.

The majority of patients presented with a single abscess (n = 52; 86.7 %), while 8 patients (13.3 %) had multiple abscesses. Lesions were multilobar in 10 cases (16.7 %). The most frequently affected cerebral regions were the frontal lobe (45.0 %), followed by the temporal (35.0 %), parietal (15.0 %), occipital (5.0 %) lobes, cerebellum (11.7 %), and brainstem including basal ganglia (10.0 %). Abscesses were localized on the left in 32 patients (53.3 %), on the right in 26 (43.3 %), and bilaterally or at the midline in 4 patients (6.7 %). The mean follow-up duration was 491.9 days, with a median of 154.5 days (Table 1).

### 3.2 Clinical outcome

Across the cohort, neurological status improved significantly following surgical intervention as reflected in all standardized outcome scales. The mean Glasgow Coma Scale (GCS) score increased from 13.4 ± 2.8 preoperatively to 14.6 ± 1.0 in the immediate postoperative period (p = 0.002). Similarly, the Karnofsky Performance Status (KPS) improved from 75.7 ± 22.7 preoperatively to 84.5 ± 17.7 immediately postoperatively (p = 0.02). The modified Rankin Scale (mRS) decreased from 2.0 ± 1.3 preoperatively to 1.1 ± 1.3 in the immediate postoperative assessment (p = 0.0001), indicating a substantial reduction in disability. The Glasgow Outcome Scale (GOS), reported exclusively as an outcome measure, demonstrated a favorable immediate postoperative score of 4.5 ± 0.7 (Table 2).

**Table 2:** Standardized neurological and functional outcome scales, MRI-based volumetrics, and follow-up outcomes of pediatric (<18 years) and adult (≥18 years) patients with primary intracerebral abscesses.

	Age <18 yrs	Age ≥18 yrs	*p-value*
***Standardized neurological and functional outcome scales***			
**GCS, mean ± SD**			
Preoperative	13.4 ± 1.7	13.4 ± 3.0	0.9
Postoperative	15.0 ± 0	14.5 ± 1.1	0.2
**GOS, mean ± SD**			
Postoperative	4.6 ± 0.7	4.4 ± 0.7	0.5
**mRS, mean ± SD**			
Preoperative	2.1 ± 1.4	2.0 ± 1.3	0.9
Postoperative	0.6 ± 0.7	1.2 ± 1.4	0.2
**KPS, mean ± SD**			
Preoperative	77.0 ± 21.6	75.4 ± 23.1	0.8
Postoperative	95.0 ± 7.1	82.4 ± 18.5	**0.04**
***Volumetrics***			
**Abscess volume (cm^3^), mean ± SD**			
Preoperative	24.0 ± 18.3	17.7 ± 15.7	0.3
Last Follow-Up	0.6 ± 1.3	2.6 ± 6.7	0.4
**Edema volume (cm^3^), mean ± SD**			
Preoperative	41.4 ± 45.2	55.8 ± 51.0	0.4
Last Follow-Up	2.7 ± 4.6	3.7 ± 9.2	0.7
***Follow-Up and outcomes***			
**Follow-Up, mean ± SD (months)**	13.3 ± 28.0	17.0 ± 22.6	0.6
**Time to second surgery/recurrence, mean ± SD (days)**	64.8 ± 93.4	19.7 ± 27.3	0.06
**Recurrence, n (%)**	6 (60.0)	20 (40.0)	0.3
**Total number of surgeries, mean ± SD**	1.8 ± 0.9	1.5 ± 0.7	0.3

GCS = Glasgow Coma Scale; GOS = Glasgow Outcome Scale; KPS = Karnofsky Performance Status Scale; mRS = modified Rankin Scale; SD = standard deviation.

Clinical symptomatology also showed marked improvement after surgery. Preoperatively, headache (51.7 %), impaired vigilance (50.0 %), and motor deficits (35.0 %) were the most frequent findings. Postoperatively, these rates declined to 3.3 %, 5.0 %, and 20.0 %, respectively. Statistically significant improvements were observed for several key symptoms, including headache (p < 0.0001), reduced vigilance (p < 0.0001), epileptic seizures (p = 0.05), infection/sepsis (p < 0.0001), vertigo (p = 0.008), nausea (p = 0.003), and aphasia (p = 0.04). Other symptoms such as paresis, sensory disturbances, and gait impairment showed trends toward improvement without reaching statistical significance. Overall, more than half of the patients (56.7 %) were asymptomatic at follow-up compared to only one patient (1.7 %) preoperatively. Of the adult patients, 52.0 % were classified as not frail (mFI-5), 28.0 % as mild frail, 16.0 % as moderate frail and 4.0 % as severely frail. No significant correlation was observed between frailty status and pre- or postoperative abscess or edema volumes (*all* p > 0.4). Among adult patients, multilobar abscess involvement correlated positively with larger abscess volume (*r* = 0.3, p = 0.03). No significant correlations were observed with body mass index (BMI) (p = 0.3) or number of abscesses (p = 0.7).

### 3.3 Volumetric outcome

**Fig. 3:**
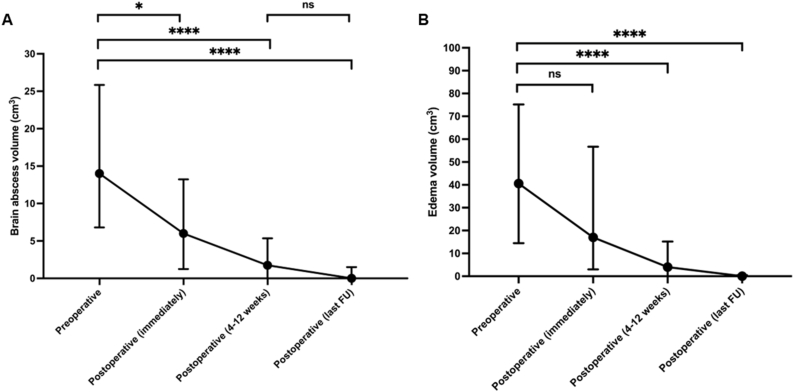
MRI-based volumetric evolution of brain abscesses and perilesional edema. A: Illustration of abscess volume reduction from preoperative to multiple postoperative time points, presented as median values with interquartile ranges (IQR). B: Illustration of edema volume decline across the same time points, likewise shown as median values with IQR, demonstrating a parallel regression pattern. Abbreviations: FU = follow-up; ns = not significant.

MRI-based volumetric analysis was performed for all 60 patients preoperatively and at multiple postoperative time points: preoperatively, immediately postoperatively (within the first five days after surgery), early follow-up (4–12 weeks postoperatively), and at the last available follow-up. The mean preoperative abscess volume was 18.8 ± 16.1 cm^3^, which decreased significantly to 10.8 ± 15.2 cm^3^ immediately after surgery (p = 0.02), 4.4 ± 6.4 cm^3^ at 4–12 weeks postoperatively (p < 0.0001), and 2.2 ± 6.3 cm^3^ at final follow-up (p < 0.0001) (Fig. 3 A). Edema volumes followed a similar pattern: 53.4 ± 50.0 cm^3^ preoperatively, declining to 35.8 ± 42.9 cm^3^ postoperatively (p = 0.06), 10.6 ± 16.8 cm^3^ at 4–12 weeks (p < 0.0001), and 3.5 ± 8.8 cm^3^ at final follow-up (p < 0.0001) (Fig. 3 B).

The relative reduction in abscess volume was similar across surgical techniques: 0.8 ± 0.3 (stereotactic aspiration), 0.9 ± 0.2 (craniotomy), and 0.9 ± 0.1 (burr-hole trepanation). No statistically significant differences were observed between groups. Although statistical comparisons including the burr-hole trepanation subgroup were performed, the limited sample size of this group necessitates cautious interpretation of these results, and these findings should be regarded as exploratory. Table 3 summarizes the volumetric measurements stratified by surgical approach.

**Table 3:** Abscess characteristics, MRI volumetrics, and antibiotic duration by surgical approach.

	stereotaxy (n = 32)	craniotomy (n = 22)	burr-hole trepanation (n = 6)	*p-value*
**Abscess characteristics**				
**Localization, n (%)**				
Frontal	15 (39.5)	10 (35.7)	2 (28.6)	0.8
Parietal	3 (7.9)	3 (10.7)	3 (42.9)	0.05
Temporal	10 (26.3)	10 (35.7)	1 (14.3)	0.6
Occipital	0	3 (10.7)	0	0.1
Brainstem	6 (15.8)	0	0	0.05
Cerebellum	4 (10.5)	2 (7.1)	1 (14.3)	0.8
**Side, n (%)**				
Right	13 (38.2)	11 (50.0)	2 (33.3)	0.6
Left	18 (47.1)	10 (45.5)	4 (66.7)	0.6
Bilateral	2 (6.2)	0	0	0.4
Midline	1 (2.9)	1 (4.5)	0	0.6
**Depth, n (%)**				
Cortical	12 (37.5)	17 (77.3)	3 (50.0)	**0.005**
Subcortical	18 (56.2)	3 (13.6)	2 (33.3)	**0.002**
Cortical + subcortical	2 (6.2)	2 (9.1)	1 (16.7)	0.7
***Volumetrics***				
**Abscess volume (cm^3^), mean ± SD**				
Preoperative	15.9 ± 14.0	21.9 ± 19.1	18.5 ± 9.9	0.4
Last Follow-Up	2.8 ± 8.1	1.5 ± 3.4	2.0 ± 2.5	0.7
**Edema volume (cm^3^), mean ± SD**				
Preoperative	50.1 ± 48.6	56.1 ± 50.9	51.6 ± 58.2	0.9
Last Follow-Up	3.9 ± 9.5	3.6 ± 8.7	0 ± 0	0.4
**Duration of antibiotics (days), mean ± SD**				
total duration	77.0 ± 39.5	59.0 ± 19.6	72.7 ± 17.1	0.1
i.v.	43.8 ± 20.7	43.0 ± 17.1	55.2 ± 17.8	0.3
p.o.	33.7 ± 38.6	16.0 ± 16.3	17.5 ± 20.6	0.3

### 3.4 Subgroup analysis: pediatric vs. adult patients

Comparison between pediatric (<18 years, n = 10) and adult patients (≥18 years, n = 50) revealed no significant differences in abscess or edema volumes at any time point. Preoperative abscess volumes were 24.0 ± 18.3 cm^3^ in children and 17.7 ± 15.7 cm^3^ in adults (p = 0.3), decreasing postoperatively to 15.0 ± 19.0 cm^3^ vs. 10.3 ± 14.3 cm^3^ (p = 0.4), then to 4.8 ± 5.7 cm^3^ vs. 4.6 ± 6.7 cm^3^ (p = 0.9), and finally to 0.6 ± 1.3 cm^3^ vs. 2.6 ± 6.7 cm^3^ (p = 0.4).

Edema volumes followed a similar trend. Preoperatively, they were 41.4 ± 45.2 cm^3^ in children and 55.8 ± 51.0 cm^3^ in adults (p = 0.3), decreasing postoperatively to 27.7 ± 35.8 cm^3^ vs. 37.9 ± 44.7 cm^3^ (p = 0.5), then to 8.3 ± 9.7 cm^3^ vs. 11.3 ± 18.1 cm^3^ (p = 0.7), and finally to 2.7 ± 4.6 cm^3^ vs. 3.7 ± 9.2 cm^3^ (p = 0.7). No statistically significant differences were observed between groups across the postoperative course.

### 3.5 Dexamethasone and volumetric correlations

Correlation analyses examined the association between dexamethasone administration and volumetric measurements of abscesses and perilesional edema at multiple timepoints: preoperatively, immediately postoperative, early follow-up (4–12 week), and at the last follow-up. Dexamethasone use was not associated with abscess volume at any imaging time point (all p > 0.05). Dexamethasone administration was associated with larger edema volumes preoperatively (p = 0.0009), immediately postoperatively (p = 0.004), and at last follow-up (p = 0.04), with a non-significant trend at 4–12 weeks (p = 0.07). Baseline perilesional edema volume was significantly larger in patients who received dexamethasone compared with those who did not (68.67 ± 52.36 cm^3^ vs. 25.10 ± 29.50 cm^3^; p = 0.0009). In univariate analysis, dexamethasone therapy (p = 0.003), dexamethasone dose (p = 0.02), and diabetes mellitus (p = 0.02) were associated with recurrence; these associations were not significant in multivariate analysis (all p > 0.05).

### 3.6 Antibiotic treatment characteristics

The mean total duration of antibiotic therapy was 69.7 ± 32.0 days, with an average intravenous course of 46.0 ± 18.3 days followed by 25.2 ± 31.0 days of oral treatment. Patients required on average 2.4 ± 1.2 changes of antibiotic regimen, reflecting adjustments for microbiological findings, tolerance, or clinical course. Importantly, the total duration of antibiotic therapy, intravenous treatment duration, and subsequent oral therapy duration did not differ significantly between the three surgical approaches, suggesting that surgical modality did not influence antimicrobial treatment length.

When stratifying patients by duration of intravenous antibiotic therapy (≤6 weeks vs. >6 weeks), no significant differences in functional outcomes were observed. Patients treated for ≤6 weeks demonstrated comparable postoperative recovery to those receiving longer therapy, with mean GCS scores of 14.7 ± 0.4 versus 14.3 ± 1.6 (p = 0.08), KPS of 85.3 ± 16.0 versus 82.4 ± 19.9 (p = 0.5), GOS of 4.6 ± 0.7 versus 4.4 ± 0.7 (p = 0.1), and mRS of 0.9 ± 1.2 versus 1.4 ± 1.4 (p = 0.1). These findings suggest that shorter courses of intravenous antibiotic therapy were not associated with inferior clinical outcomes.

### 3.7 Recurrence and secondary surgical procedures

Several patients required re-operation due to persistent or recurrent abscess formation. In total, 26 revision procedures were performed in 26 patients, comprising 15 stereotactic aspirations, 11 craniotomies, and 1 burr-hole trepanation (Table 4). Among those who underwent revision surgery, 46.9 % had initially been treated with stereotactic aspiration, 45.4 % with craniotomy, and 16.7 % with burr-hole trepanation. These percentages reflect the distribution of surgical approaches among all patients who required a second intervention, and no statistically significant differences were observed between groups.

**Table 4:** Comparison of characteristics between recurrent and non-recurrent primary brain abscesses.

	recurrences (n = 26)	no recurrences (n = 34)	*p-value*
**Abscess characteristics**			
**Initial surgical approach, n (%)**			
Stereotaxy	15 (57.7)	17 (50.0)	0.6
Craniotomy	10 (38.5)	12 (35.3)	0.99
Burr-hole trepanation	1 (3.8)	5 (14.7)	0.2
**Localization, n (%)**			
Frontal	15 (44.1)	12 (30.8)	0.3
Parietal	4 (11.8)	5 (12.8)	0.99
Temporal	12 (35.3)	9 (23.1)	0.3
Occipital	0	3 (7.7)	0.2
Brainstem	0	6 (15.4)	**0.03**
Cerebellum	3 (8.8)	4 (10.3)	0.99
**Side, n (%)**			
Right	10 (37.0)	16 (45.7)	0.6
Left	14 (51.9)	18 (51.4)	0.99
Bilateral	2 (7.4)	0	0.2
Midline	1 (3.7)	1 (2.9)	0.99
**Depth, n (%)**			
Cortical	15 (57.7)	17 (50.0)	0.6
Subcortical	8 (30.8)	15 (44.1)	0.4
Cortical + subcortical	3 (11.5)	2 (5.9)	0.6
***Volumetrics***			
**Abscess volume (cm^3^), mean ± SD**			
Preoperative	22.9 ± 15.2	15.5 ± 16.3	0.08
Last Follow-Up	1.8 ± 4.4	2.6 ± 7.5	0.6
**Edema volume (cm^3^), mean ± SD**			
Preoperative	59.5 ± 48.4	48.8 ± 51.5	0.4
Last Follow-Up	3.4 ± 6.3	3.5 ± 10.5	0.9

**Fig. 4:**
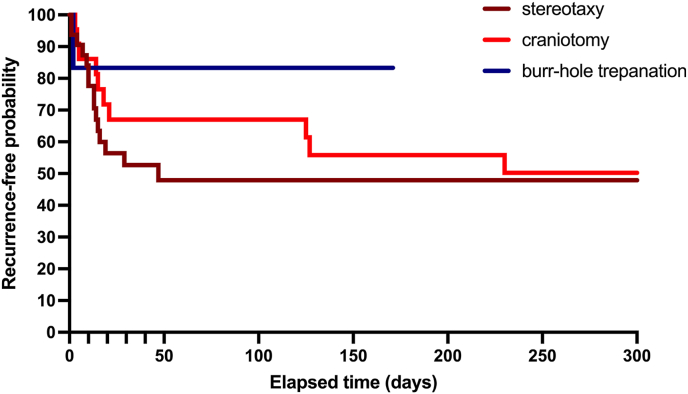
Kaplan–Meier analysis of time to secondary surgical intervention. The figure depicts the recurrence-free probability stratified by surgical approach (stereotactic aspiration, craniotomy, burr-hole trepanation).

The timing of secondary procedure varied according to surgical modality (Fig. 4). The mean interval to second surgery was 13.9 ± 11.6 days following stereotactic aspiration, 56.2 ± 77.7 days after craniotomy, and 2 days in the single case treated with burr-hole trepanation. Pairwise comparisons revealed no significant differences between surgical approaches (stereotaxy vs. craniotomy: p = 0.5; stereotaxy vs. burr-hole trepanation: p = 0.3; burr-hole trepanation vs. craniotomy: p = 0.5). No meaningful clinical or volumetric differences were observed between abscesses that recurred and those that did not require further surgery. While univariate analysis suggested an association of recurrence with diabetes mellitus (p = 0.02) and extended dexamethasone administration (p = 0.02), these findings were not validated in the multivariate analysis.

### 3.8 Microbiological findings and pathogen distribution

Microbiological analysis across all cases revealed a cumulative total of 92 pathogens, with 24 distinct species identified, as several abscesses yielded multiple isolates, highlighting the polymicrobial nature of brain abscesses. Frequently detected organisms were Streptococcus spp. (37.0 %), Fusobacterium spp. (14.1 %), *Aggregatibacter aphrophilus* (8.7 %), *Staphylococcus aureus* (2.2 %) and other staphylococci (5.4 %) (Table 5).

**Table 5:** Microbiological findings in brain abscesses. A total of 24 distinct pathogens were identified, with a cumulative 92 isolates across all cases, reflecting the polymicrobial nature of some abscesses. Number of detections (n) and corresponding percentages (%) are shown for each pathogen.

Pathogen	n	%
Streptococcus spp.	34	37.0 %
Fusobacterium spp.	13	14.1 %
*Staphylococcus aureus*	2	2.2 %
Other staphylococci	5	5.4 %
*Aggregatibacter aphrophilus*	8	8.7 %
*Parvimonas micra*	4	4.3 %
Nocardia spp.	3	3.3 %
*E. coli*	2	2.2 %
Cutibacterium acnes	2	2.2 %
*Prevotella oris*	2	2.2 %
*Bacteroides fragilis*	2	2.2 %
*Aspergillus fumigatus*	2	2.2 %
Toxoplasma gondii	2	2.2 %
*Listeria monocytogenes*	1	1.1 %
*Eikenella corrodens*	1	1.1 %
*Haemophilus haemolyticus*	1	1.1 %
*Haemophilus influenzae*	1	1.1 %
*Citrobacter koseri*	1	1.1 %
*Proteus mirabilis*	1	1.1 %
*Pseudomonas aeruginosa*	1	1.1 %
*Peptoniphilus indolicus*	1	1.1 %
*Filifactor alocis*	1	1.1 %
*Dialister pneumosintes*	1	1.1 %
Scedosporium apiospermum	1	1.1 %

## 4 Discussion

This retrospective study compares clinical and radiological outcomes of stereotactic aspiration, craniotomy, and burr-hole trepanation in primary intracerebral abscesses. All three approaches yielded significant and sustained reductions in abscess and perilesional edema volumes, with comparable long-term neurological recovery in both adults and children. A novel aspect of this work is the systematic volumetric MRI analysis at defined pre- and postoperative time points, providing new insights into the temporal evolution of abscess and edema resolution – an area not yet addressed in previous research.

Across the cohort, abscess volumes demonstrated a progressive reduction over postoperative follow-up, with comparable volumetric trajectories among surgical approaches, although revision surgery was required in some patients due to insufficient early resolution or recurrence. This aligns with prior studies reporting the efficacy of both minimally invasive and open surgical methods in abscess evacuation (Ali et al., 2023; Tan et al., 2010). The similar patterns of volumetric reduction observed after stereotactic aspiration and craniotomy suggest comparable radiological trajectories in appropriately selected patients, while acknowledging that these findings arise from a retrospective, non-randomized cohort and do not necessarily establish equivalence between techniques.

The observed distribution of surgical techniques – craniotomy for cortical abscesses and stereotactic aspiration for deeper lesions – appears to mirror routine neurosurgical practice driven by lesion accessibility and safety considerations, rather than indicating comparative superiority of either approach (Table 3).

The role of dexamethasone in the management of brain abscesses remains controversial. While its use is common in clinical practice to reduce cerebral edema and mass effect, definitive evidence for its benefit is lacking. A systematic meta-analysis demonstrated that the addition of dexamethasone to the treatment regimen for patients with brain abscesses was not associated with increased mortality, suggesting that its use may be safe in selected cases (Simjian et al., 2018). However, the decision to administer dexamethasone remains at the discretion of the treating physician, given the absence of clear, evidence-based guidelines. The observed association between dexamethasone administration and larger edema volumes in our study reflects confounding by indication, as corticosteroids were preferentially prescribed in patients with pronounced mass effect and more extensive radiological edema. Given the retrospective design and the absence of baseline comparability between treated and untreated patients, no causal inference regarding the effect of dexamethasone on edema evolution, abscess resolution, or clinical outcome can be drawn from the present data. Accordingly, corticosteroid use in this cohort should be interpreted solely as a marker of disease severity rather than a determinant of radiological or functional outcome.

Importantly, the absence of significant differences in abscess and edema volumes or clinical outcomes between pediatric and adult patients suggests similar pathophysiological responses and treatment efficacy across age groups.

Our analysis did not reveal correlations between frailty status and volumetric or neurological outcomes. This discrepancy may be partly explained by the relatively young median age of our patient cohort (mean age 48.0 years), resulting in fewer frail individuals and limited variability in frailty scores. Consequently, the Modified 5-Item Frailty Index (mFI-5) may have limited sensitivity and applicability in this comparatively young and less comorbid population, reducing its utility as a prognostic tool in our primary intracerebral abscess patients.

Recurrence was not clearly linked to surgical technique, and the need for revision surgery in some patients highlights the importance of continued clinical and radiological surveillance.

**Fig. 5:**
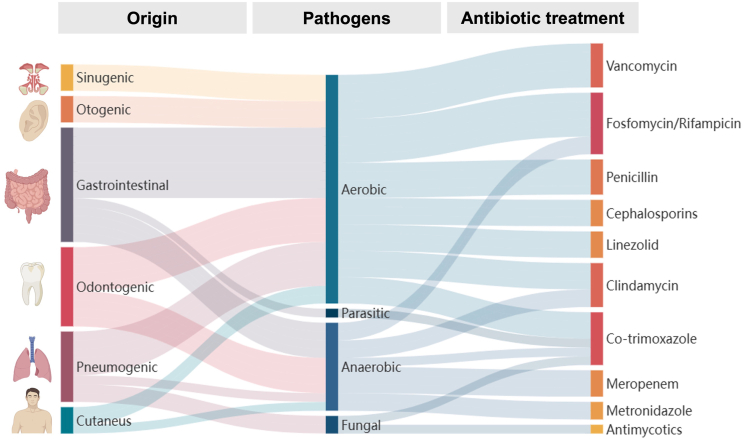
Sankey diagram depicting the general relationships between presumed infectious foci of brain abscesses, associated pathogen classes, and potential antimicrobial treatment options. This visualization highlights the multifactorial etiology of brain abscesses and the complexity of pathogen-directed therapy.

The optimal duration of intravenous antibiotic therapy for brain abscesses remains debated. The European Society of Clinical Microbiology and Infectious Diseases (ESCMID) conditionally advises 6–8 weeks of intravenous treatment for brain abscesses managed by aspiration or conservative means (Edwards-Bailey et al., 2025; Lu et al., 2006; Bodilsen et al., 2024). Prolonged antimicrobial exposure, however, carries considerable risks, including drug-induced neutropenia and other treatment-related toxicities as well as selection pressure and development of resistance. At present, the ORAL trial (“Partial oral antibiotic treatment for bacterial brain abscess: an open-label randomized non-inferiority trial”) is underway (Bodilsen et al., 2021), investigating whether an early switch to oral therapy after at least two weeks of intravenous treatment is non-inferior to the conventional standard of six weeks or longer of intravenous antibiotics. In our cohort, the mean intravenous treatment duration was 46.0 ± 18.3 days, aligning with current guidelines and proving safe with favorable outcomes. Notably, our patients receiving less than 6 weeks of intravenous antibiotic therapy showed similarly improved functional outcomes, suggesting shorter courses may be feasible. The parallel volumetric regression of abscess and edema further highlights serial MRI as a potential biomarker to guide individualized antibiotic duration and identify candidates for earlier transition to oral therapy (Bodilsen et al., 2023). To better contextualize the microbiological dimension of brain abscesses, we also provide a Sankey diagram (Fig. 5) illustrating the general relationships between presumed infectious foci, associated pathogen classes, and potential antimicrobial treatment strategies.

One of the primary strengths of our study lies in its detailed MRI-based volumetric assessment of abscess and perilesional edema at multiple postoperative time points. To our knowledge, this is the only study to characterize the temporal evolution of both abscess size and brain edema longitudinally in a clinical cohort. This approach provides valuable insights into the healing trajectory, facilitating more precise monitoring and potentially enabling earlier identification of patients at risk for delayed recovery or complications. One of the central clinical challenges in the management of intracerebral abscesses is determining the expected pace of radiological resolution under successful therapy and identifying early deviations that should prompt reconsideration of the treatment strategy. Although the present dataset does not allow for fixed-timepoint modeling of abscess and edema resolution, the longitudinal volumetric trajectories depicted in Fig. 3 provide a pragmatic framework for clinical interpretation. In patients with favorable outcomes, abscess and edema volumes demonstrated a consistent downward trend over time. Conversely, the need for surgical revision was predominantly driven by an early lack of volumetric regression, most often within the first postoperative month. In this context, persistent or increasing lesion volumes during early follow-up represented a practical red flag in routine clinical care, frequently leading to re-aspiration or alternative surgical management.

We minimized bias by including all consecutive eligible patients, applying strict inclusion criteria, using standardized volumetric imaging protocols, and assessing outcomes with validated clinical scales. Data were reviewed independently of treating physicians, and only patients with complete follow-up were analyzed.

Nevertheless, several limitations warrant consideration. The retrospective design inherently limits causal inference and introduces selection bias, particularly regarding the non-randomized choice of surgical technique based on clinical judgement and the absence of standardized clinical criteria for surgical technique selection. Thus, comparisons between surgical approaches are influenced by indication bias related to surgeon-selected treatment allocation and should therefore be interpreted as descriptive observations rather than evidence of causal differences between techniques. Furthermore, given the retrospective design, heterogeneous follow-up intervals, and unequal subgroup sizes, volumetric changes can only be reported as descriptive longitudinal trajectories rather than statistically modeled rates of reduction. Accordingly, the present findings should be interpreted as descriptive comparisons of longitudinal volumetric evolution rather than as evidence of statistically modeled equivalence or superiority of abscess reduction rates between surgical approaches. The extremely limited sample size of the burr-hole trepanation subgroup substantially restricts statistical power and precludes reliable comparative conclusions regarding this surgical approach and reduces the generalizability of subgroup analyses. Variations in MRI acquisition protocols and scanner types over the study period could have affected volumetric measurements despite standardized segmentation procedures. In addition, the absence of an observed association between frailty and clinical or volumetric outcomes likely reflects limited applicability and sensitivity of the mFI-5 in this relatively young and low-frailty cohort, rather than evidence against a role of frailty in brain abscess outcomes. Moreover, any observed association between dexamethasone administration and edema volume in this cohort is subject to substantial confounding by indication, precluding causal inference regarding corticosteroid effects on radiological or clinical outcomes. Finally, detailed data on immunosuppressive status were limited, which might influence abscess resolution and clinical outcomes.

A comparison with conservatively treated patients was not included, as these individuals represent a fundamentally different clinical population characterized by smaller lesion size, deep or eloquent location, limited mass effect, or prohibitive comorbidities. Such selection introduces substantial confounding by indication and is further compounded by non-standardized and potentially less frequent imaging follow-up in non-surgical management. Consequently, volumetric trajectories in conservatively treated cases are neither biologically nor methodologically comparable to postoperative volumetric evolution, and inclusion of such a group would risk misleading interpretation rather than strengthen the analysis.

In conclusion, stereotactic aspiration, craniotomy, and burr-hole trepanation are all effective for managing primary intracerebral abscesses, achieving comparable reductions in abscess and edema volumes with favorable long-term outcomes. Serial MRI volumetrics provide a valuable means of monitoring treatment response, and future multicenter studies with larger cohorts and standardized imaging are needed to confirm these results and optimize surgical strategies.

## 5 Conclusions

In summary, this retrospective study demonstrates that contemporary surgical management of primary intracerebral abscesses is associated with favorable radiological and functional outcomes across commonly used surgical approaches: stereotactic aspiration, open craniotomy, and burr-hole trepanation. To our knowledge, this is one of the few studies to analyze brain abscesses with such detailed focus on volumetric changes of both abscess and perilesional edema over the course of treatment. Our results demonstrate that surgical treatment not only achieved a sustained radiological resolution of both the abscess cavity and the associated perilesional edema, but that these structural improvements were paralleled by meaningful clinical recovery. Serial MRI-based volumetric assessment emerges as a practical tool for postoperative monitoring, offering objective insight into treatment response and early identification of atypical healing trajectories. These findings contribute to a more differentiated understanding of therapeutic strategies and provides detailed data on the expected volumetric resolution trajectory in primary brain abscesses, which may aid in postoperative monitoring and the timing of follow-up imaging. While these findings support the clinical utility of longitudinal imaging in routine care, they should be interpreted within the constraints of a retrospective, non-randomized design, underscoring the need for prospective, standardized studies to refine surgical decision-making and postoperative surveillance. Routine incorporation of serial MRI-based volumetric assessment into postoperative follow-up may enable earlier detection of insufficient lesion regression, thereby supporting timely treatment adjustment and more individualized management of patients with primary brain abscesses.

## Declaration of competing interest

The authors declare that they have no known competing financial interests or personal relationships that could have appeared to influence the work reported in this paper.
